# Supplementary material for: A targeted amplicon sequencing panel for cost-effective high-throughput genotyping of Aedes aegypti
Source: bioRxiv. 2025 Sep 30:2025.09.28.679043. Preprint. [Version 1] doi: 10.1101/2025.09.28.679043 (PMC12621932; doi:10.1101/2025.09.28.679043)
Supplement: 3 [file NIHPP2025.09.28.679043v1-supplement-3.pdf]

bioRxiv preprint doi: https://doi.org/10.1101/2025.09.28.679043; this version posted September 30, 2025. The copyright holder for this preprint (which was not certified by peer review) is the author/funder, who has granted bioRxiv a license to display the preprint in perpetuity. It is made available under aCC-BY 4.0 International license.

Supplementary figures:

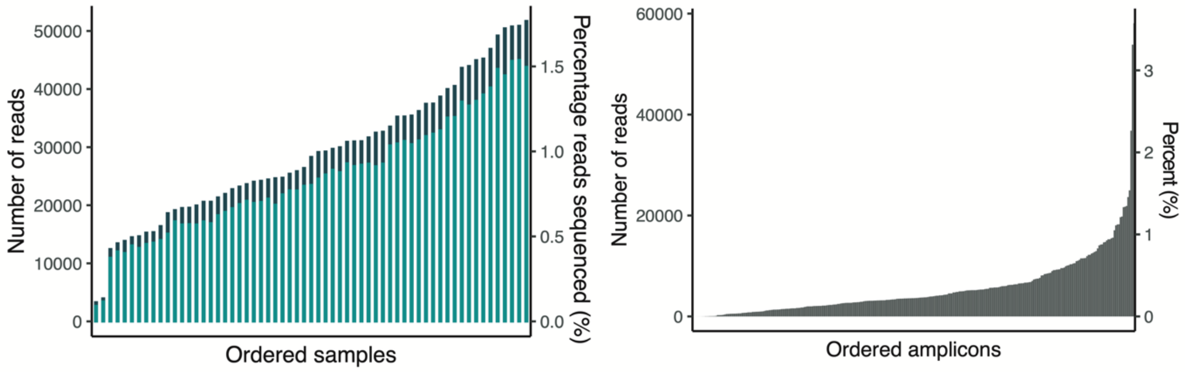

Figure S1: Mapping statistics of the technical replicate run. (**A**) Number of reads per sample mapped to the amplicon loci (light green) or the AaegL5 assembly (dark green), respectively. (**B**) Number of reads uniquely mapping to each amplicon, ordered by increasing coverage.

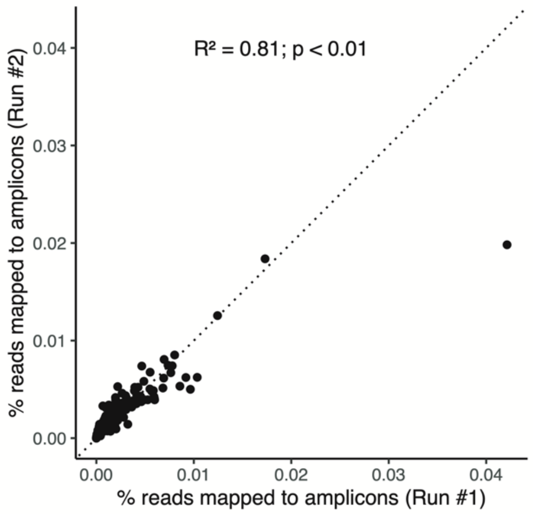

Figure S2. Amplicon sequencing reproducibility. Correlation between the percentage of reads mapped to each amplicon locus in the two replicate sequencing runs.

bioRxiv preprint doi: https://doi.org/10.1101/2025.09.28.679043; this version posted September 30, 2025. The copyright holder for this preprint (which was not certified by peer review) is the author/funder, who has granted bioRxiv a license to display the preprint in perpetuity. It is made available under aCC-BY 4.0 International license.

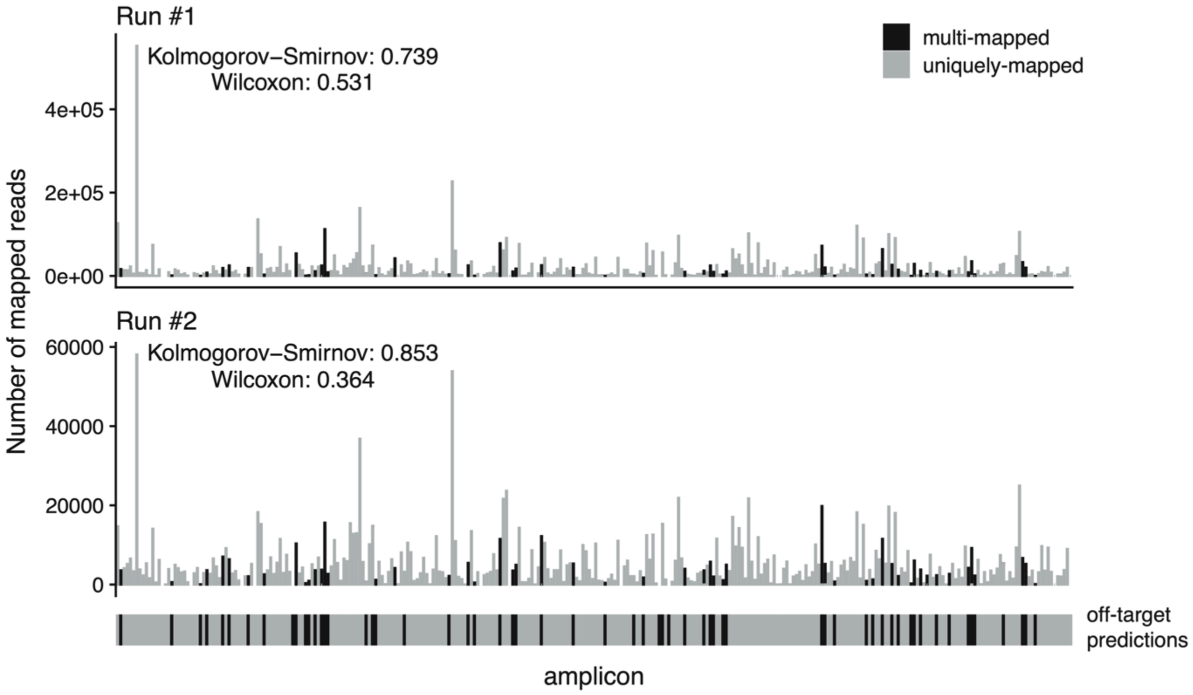

Figure S3: Visualization of amplicons with putative off-target amplification. The bottom panel shows the 291 amplicons, with vertical black lines indicating primer pairs that matched multiple sites in the genome. The top panel shows the number of reads mapped to each amplicon; gray bars represent uniquely mapped reads, while black lines represent multi-mapped reads. The Kolmogorov-Smirnov and Wilcoxon tests were conducted to assess whether there was a difference in the number of mapped reads between amplicons with predicted off-target loci and the other amplicons.

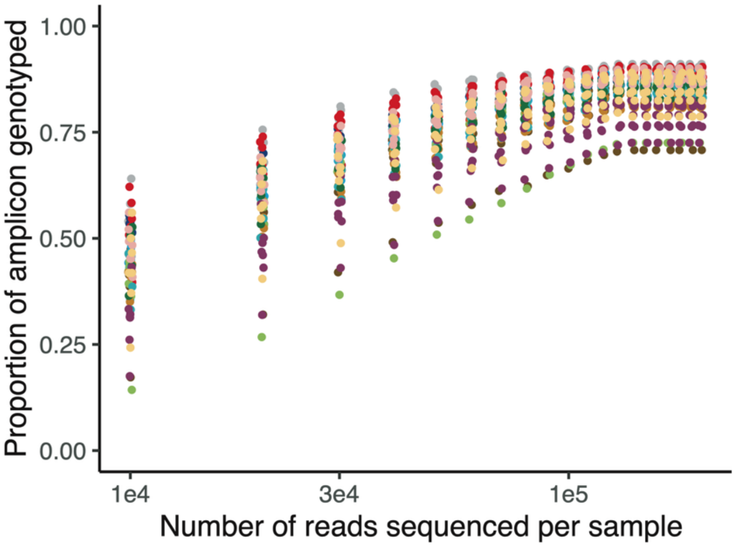

Figure S4: Rarefaction analysis of the relationship between the amplicon coverage and sequencing effort. The graph shows the proportion of amplicons genotyped (>10× coverage) as a function of the read counts per sample. This analysis was performed by computationally resampling from 1 to 20 million reads using data from a sequencing run that yielded about 8 million reads. Different colors represent individual samples.

bioRxiv preprint doi: https://doi.org/10.1101/2025.09.28.679043; this version posted September 30, 2025. The copyright holder for this preprint (which was not certified by peer review) is the author/funder, who has granted bioRxiv a license to display the preprint in perpetuity. It is made available under aCC-BY 4.0 International license.

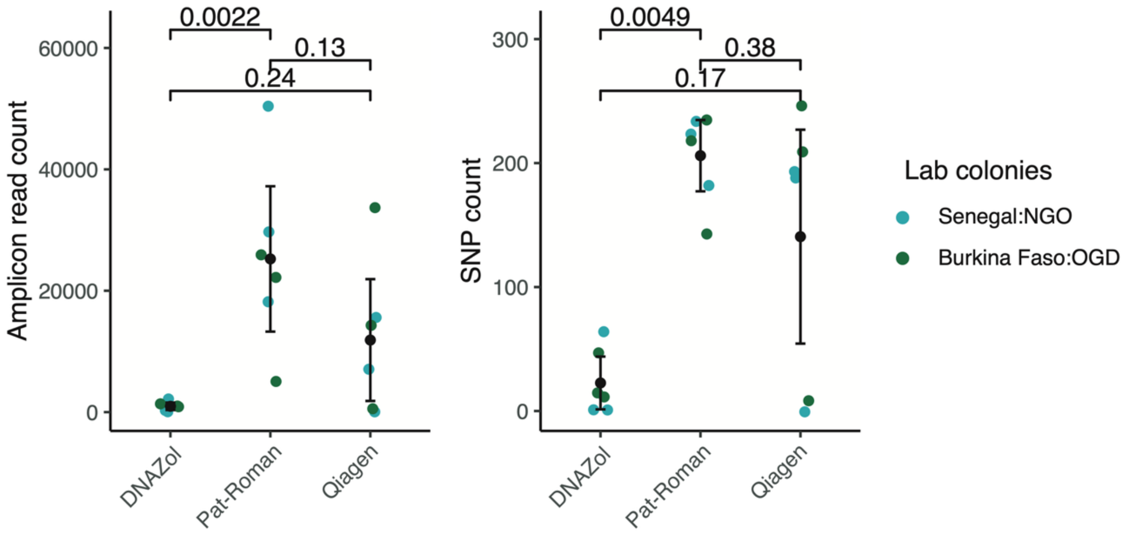

Figure S5. Comparison of three DNA extraction methods. Two commercial kits (Qiagen DNeasy Blood & Tissue and Molecular Research Center Inc. DNAzol DIRECT) and a low-cost homemade method (Pat-Roman) were compared based on the number of reads and SNPs genotyped from the same set of samples processed in the same library. The left panel shows the number of reads mapped to amplicon loci per sample, whereas the right panel displays the total number of SNPs identified. The data points are individual samples color-coded by colony, and the black vertical bar indicates the mean and 95% confidence interval. The p-values above the graphs were obtained using Wilcoxon test.

Supplementary table legends:

Table S1: Amplicon loci and primers. For each amplicon, the table provides the genomic positions, primer sequences, annealing temperature (Tm), and balancing factors.

Table S2: Cost estimates. The cost per sample was estimated based on the current price list at Institut Pasteur (France) and Yale University (United States). The number of samples per Illumina MiSeq Micro flowcell (n=80) was set to obtain 100,000 reads per sample, which we identified as the optimal read coverage based on the rarefaction analysis.
